# Inflammatory links between epilepsy and depression: a review of mechanisms and therapeutic strategies

**DOI:** 10.3389/fnins.2025.1614297

**Published:** 2025-06-26

**Authors:** Yu-Qian Shi, He-Cai Yang, Cong He, Yu-Hao Wang, Jia Zheng, Xing-Yi Wang, Fang-Yi Hao, Chu-Wen Feng, Lin Ma, Yue-Hui Zhang, Zheng Liu

**Affiliations:** ^1^First Clinical Medical College, Heilongjiang University of Chinese Medicine, Harbin, China; ^2^The Second Affiliated Hospital of Heilongjiang University of Chinese Medicine, Harbin, China; ^3^The First Affiliated Hospital of Heilongjiang University of Chinese Medicine, Harbin, China

**Keywords:** epilepsy-depression comorbidity, neuroinflammation, gut-brain axis, natural anti-inflammatory agents, aging-related inflammation

## Abstract

Depression is the most common psychiatric comorbidity of epilepsy. While antiseizure medications (ASMs) can exacerbate depressive symptoms, depression itself may increase both the frequency and duration of epileptic seizures. The pathophysiological mechanisms underlying epilepsy-depression comorbidity (EDC) involve neurotransmitter imbalance, inflammatory responses, oxidative stress (OS), gut microbiota dysbiosis, and neuroendocrine abnormalities. Recent studies highlight that inflammation contributes to EDC via multiple interconnected mechanisms, including glial cell activation, cytokine release, pyroptosis, and oxidative damage, ultimately leading to synaptic dysfunction and neuroimmune imbalance. Drawing from representative and recent evidence, this review summarizes the role of inflammation in the pathogenesis and progression of EDC. It also outlines current inflammation-targeted therapeutic strategies, such as anti-inflammatory drugs, antioxidants, herbal medicine, acupuncture, probiotic modulation, and precision therapies. This review provides a conceptual framework for understanding inflammation-mediated EDC and offers insights into targeted treatment approaches.

## 1 Introduction

Epilepsy is a common disorder of the central nervous system (CNS), characterized not only by recurrent epileptic discharges but also by frequent and severe neuropsychiatric comorbidities, with depression being the most prevalent (Alhashimi et al., 2022). According to updated global estimates, approximately 24.22 million individuals were living with epilepsy as of 2021, with an age-standardized incidence rate of 42.8 per 100,000 population. The incidence was notably highest among children aged 0–14 years, reaching 61 per 100,000 (Fang et al., 2025). During the same period, China’s epilepsy prevalence and incidence reached 214.7 and 28.2 per 100,000, respectively, reflecting increases of 13.4 and 26.1% from 1990 levels. In contrast, the age-standardized mortality rate declined by over 50% (Vayanakkan et al., 2024). A nationwide register-based cohort study involving over 8.7 million individuals in Denmark demonstrated a long-term bidirectional relationship between epilepsy and depression. Specifically, individuals diagnosed with unipolar depression had a significantly elevated risk of subsequently developing epilepsy, with an adjusted hazard ratio (aHR) of 2.35 (95% CI: 2.25–2.44), even after adjustment for comorbidities, substance abuse, and calendar year. Conversely, individuals with epilepsy also had a higher risk of developing depression (aHR = 1.88, 95% CI: 1.82–1.95); this association remained robust across age groups and throughout the follow-up period (Bølling-Ladegaard et al., 2023). These findings suggest that epilepsy and depression are not merely co-occurring by chance but may share underlying pathophysiological mechanisms (Kanner, 2003; Tang et al., 2022). Given the high prevalence and significant adverse impact of epilepsy-depression comorbidity (EDC), exploring its underlying mechanisms and identifying effective intervention strategies is crucial.

Current studies suggest that the pathophysiological mechanisms of EDC involve multiple factors, including chronic inflammation, neurotransmitter imbalance, hypothalamic–pituitary–adrenal (HPA) axis dysfunction, and impaired synaptic plasticity (Yan et al., 2025). Among these factors, inflammation is considered a central mediator of the bidirectional relationship between epilepsy and depression. Mazarati et al. (2017) demonstrated that elevated inflammatory cytokines are a shared feature of both epilepsy and depression. Persistent neuroinflammation may disrupt the 5-hydroxytryptamine (5-HT) signaling pathway, exacerbate neural dysfunction, and, through a positive feedback mechanism, impair neural circuit remodeling and synaptic plasticity, thereby contributing to the comorbid progression of both disorders.

Beyond these endogenous pathophysiological mechanisms, antiseizure medications (ASMs), the cornerstone of epilepsy treatment, also exert complex modulatory influences on affective states, significantly contributing to the clinical presentation and management of EDC. Certain classical ASMs (e.g., phenobarbital, benzodiazepines, topiramate, vigabatrin) primarily act by broadly suppressing neuronal excitability or markedly enhancing gamma-aminobutyric acid (GABA) receptor activity. While effective for seizure control, these actions are frequently associated with adverse emotional sequelae, including emotional blunting, apathy, and psychomotor/cognitive slowing, particularly in predisposed individuals. Disturbances in folate metabolism or sleep architecture can further potentiate these psychiatric side effects (Mula and Sander, 2007). Certain classical ASMs (e.g., phenobarbital, benzodiazepines, topiramate, vigabatrin) primarily act by broadly suppressing neuronal excitability or markedly enhancing GABA receptor activity. While effective for seizure control, these actions are frequently associated with adverse emotional sequelae, including emotional blunting, apathy, and psychomotor/cognitive slowing, particularly in predisposed individuals; factors such as disturbances in folate metabolism or sleep architecture can further potentiate these psychiatric side effects (Mula and Sander, 2007). Conversely, other ASMs (e.g., lamotrigine, valproate, carbamazepine, gabapentin) are associated with more favorable mood profiles, with some demonstrating inherent mood-stabilizing or even antidepressant properties. Their beneficial mechanisms can include the attenuation of neuroinflammation [e.g., valproate via histone deacetylase (HDAC) inhibition in microglia, downregulating interleukin-1 beta (IL-1β) and inducible nitric oxide synthase (iNOS)] or the mitigation of glutamatergic excitotoxicity coupled with enhancement of neurotrophic factor expression (e.g., lamotrigine), thereby conferring antidepressant-like potential (Mula and Sander, 2007; Chen et al., 2007). Newer-generation ASMs like levetiracetam (LEV) present more nuanced neuropsychiatric profiles. Although occasionally linked to irritability or agitation, mechanistic investigations also suggest LEV can mitigate neuroinflammation, for instance, by suppressing transcription factors and restoring astrocytic gap junction coupling, potentially contributing to improved affect regulation (Kim et al., 2010; Mohammad et al., 2019). Collectively, these observations underscore that the distinct psychotropic profiles of ASMs are crucial considerations in the clinical management of EDC. The rational selection of pharmacotherapeutic agents that effectively control seizures while exerting minimal adverse—or ideally, beneficial—effects on mood, including potential anti-inflammatory actions, is essential for developing individualized, integrative treatment strategies. Such strategies must comprehensively address both the neurological and psychiatric dimensions inherent to this complex comorbid condition.

In recent years, inflammation-targeted therapeutic strategies have gained increasing traction in the fields of epilepsy and psychiatric disorders. Cai et al. (2024) reported that epileptic seizures can induce microglial activation and trigger a cascade involving the NOD-, LRR-and pyrin domain-containing protein 3 (NLRP3) inflammasome, leading to elevated pro-inflammatory cytokine levels, aggravated neuronal damage, and depression-like behaviors. Further investigations revealed that AM1241, a selective cannabinoid receptor type 2 (CB2) agonist, can inhibit pro-inflammatory polarization of microglia and reduce NLRP3-mediated neuroinflammation, thereby attenuating seizure frequency and alleviating depressive symptoms.

Based on these findings, this review summarizes the pathophysiological mechanisms underlying EDC, with a particular focus on how chronic inflammation contributes to its development and progression through pathways involving neurotransmitter imbalance, HPA axis dysregulation, and impaired synaptic plasticity. Furthermore, current potential therapeutic strategies targeting inflammatory pathways are reviewed, including anti-inflammatory agents (e.g., cyclooxygenase-2 (COX-2) and NLRP3 inhibitors), antioxidants (such as N-acetylcysteine (NAC) and melatonin), probiotic modulation (via gut-brain axis (GBA) regulation), and precision therapies (such as mesenchymal stem cells (MSCs) and exosome-based approaches).

## 2 PART 1. Association between epilepsy and depression

Epilepsy and depression share complex comorbidity mechanisms, involving factors such as seizure focus localization, sex differences, genetic susceptibility, and specific structural and functional brain alterations, including disruptions in emotion-regulation neural circuits (e.g., involving the hippocampus, amygdala, and prefrontal cortex), changes in gray and white matter integrity, and altered functional connectivity.

### 2.1 Seizure focus localization: high comorbidity between temporal lobe epilepsy and affective disorders

TLE is more likely than other types of epilepsy to be accompanied by psychiatric and affective disorders. Lu et al. (2021) found that the incidence of depression in patients with TLE reached 53.8%, significantly higher than the 25% observed in patients with extratemporal epilepsy. Recent studies (Vinti et al., 2021; Wen et al., 2024) have further indicated that patients with TLE have the highest risk of comorbid psychiatric disorders, particularly depression and anxiety. This may be closely associated with emotional dysregulation caused by limbic system involvement, such as the hippocampus and amygdala. Psychiatric symptoms in TLE patients are also linked to the International Classification of Cognitive and Behavioral Comorbidities of Epilepsy. Patients with the temporal lobe epilepsy-depression (TLE-D) phenotype are more prone to cognitive impairment, a finding that further supports the notion that TLE may affect both cognitive and emotional functions through multiple pathophysiological mechanisms (Bingaman et al., 2023).

### 2.2 Sex differences: increased risk of depression in female patients with epilepsy

Epidemiological data indicate that female patients with epilepsy are more likely than males to develop depression. Studies (Guo et al., 2023) have shown that the incidence of depression in females is approximately 4.27 times higher than in males, which may be related to psychosocial stress, hormonal fluctuations, and physiological factors. Recent studies by Ogunjimi et al. (2024) have identified close association between dysregulated fluctuations in sex steroid hormone levels, such as prolactin and testosterone, and the pathophysiology of EDC. Compared to males, females are more susceptible to physiological events such as menstruation, pregnancy, and menopause, which lead to greater hormonal variability and may contribute to the higher prevalence of EDC in women. These findings suggest that endocrine regulation may play a pivotal role in the sex-specific vulnerability to EDC.

### 2.3 Genetic susceptibility: shared genetic architecture

EDC exhibits significant genetic susceptibility, with its comorbidity risk potentially influenced by shared genetic variants. Studies have shown that first-degree relatives of individuals with epilepsy or depression have a significantly increased risk of comorbidity, suggesting a role of familial genetic factors in the development of EDC (Shaikh et al., 2020). Large-scale genomic analyses have further revealed cross-trait genetic enrichment between generalized genetic epilepsy and depression (Karadag et al., 2023). Epilepsy also shares multiple genetic risk loci with other neuropsychiatric disorders, including schizophrenia and bipolar disorder. Genes such as *VRK*_2_, *PTPRK*, and *ZSCAN*_23_ may increase individual susceptibility to EDC by modulating neurodevelopment, synaptic plasticity, and neuroimmune pathways (Struck et al., 2021). These findings suggest that EDC may be influenced by complex polygenic regulation, and its genetic mechanisms warrant further investigation.

### 2.4 Structural and functional brain abnormalities: the central role of neural circuit disruption

Structural and functional brain abnormalities are also involved in the pathogenesis and progression of EDC, including disruptions in neural circuits, alterations in gray matter volume, and abnormalities in white matter microstructure. Neuroimaging studies (Wen et al., 2024) have shown that patients with EDC often exhibit hippocampal atrophy, amygdalar volume abnormalities, and disrupted prefrontal-limbic connectivity, indicating impairment of emotion-regulation-related neural networks. Compared to patients with TLE, those with the TLE-D phenotype exhibit more extensive structural and functional brain abnormalities, including reduced gray matter volume in cortical and subcortical regions, cerebellar damage, and white matter microstructural changes. With advances in neuroimaging and molecular techniques, significant differences in brain metabolism and functional activity have been identified in TLE patients with comorbid depression. Fluorodeoxyglucose-Positron Emission Tomography (FDG-PET) studies have shown widespread reductions in cerebral metabolism, while functional Magnetic Resonance Imaging (fMRI) studies have revealed more pronounced alterations in functional brain networks in TLE patients with depression (Struck et al., 2021). These findings suggest that structural brain abnormalities not only serve as biomarkers of EDC but also influence emotional regulation and disease progression.

## 3 PART 2. Mechanistic role of inflammation in EDC

Inflammation is considered one of the fundamental pathophysiological mechanisms underlying EDC. Epileptic seizures can activate the HPA axis through inflammatory pathways involving interleukin-1β (IL-1β) and nuclear factor-kappa B (NF-κB), resulting in elevated cortisol levels and increased risk of depression (Mukhtar, 2025). Concurrently, patients with depression commonly exhibit chronic low-grade inflammation, characterized by persistently elevated pro-inflammatory cytokines [e.g., interleukin-6 (IL-6), Tumor Necrosis Factor-alpha (TNF-α)] and excessive activation of glial cells (Vayanakkan et al., 2024). Inflammation is not only a shared pathophysiological feature of epilepsy and depression but may also serve as a mechanistic bridge linking the two conditions. For example, proinflammatory cytokines such as IL-1β have been shown to impair neurotransmitter homeostasis (e.g., decreased serotonin levels and enhanced glutamatergic excitotoxicity), induce oxidative stress (OS) and neuronal pyroptosis, and lead to mitochondrial dysfunction and synaptic protein loss—ultimately fostering a bidirectional relationship between epilepsy and depression (Zhu et al., 2006; Tong et al., 2024).

This section explores the role of inflammation in the pathogenesis and progression of EDC. As shown in Table 1, this section reviews recent advances in understanding the role of inflammation in EDC, focusing on evidence that inflammation affects the EDC disease course through multiple mechanisms, including neurotransmitter homeostasis, HPA axis regulation, immune cell activation, pyroptosis and OS pathways, and synaptic plasticity.

**Table 1:** Summary of inflammation-related mechanistic evidence in studies of EDC.

References	Study type	Model description	Inflammatory cytokines↑	Cytokines	Neurotransmitter abnormalities	HPA axis activation	Immune cell activation	Cell types activated	Pyroptosis/oxidative stress	Synaptic remodeling	Anti-inflammatory or antioxidant intervention	Behavioral assessment
Tong et al. (2024)	Human + animal	Epilepsy patients and SE rat model (PILO-induced)	✓	IL-18, IL-1β	–	–	–	Neurons, microglia	✓	–	✓	✓
Deng et al. (2021)	Human + animal	Epilepsy patients and status epilepticus (SE) rat model	✓	IL-1β, IL-6, TNF-α	–	–	✓	Microglia, astrocytes	✓	–	–	✓
Younis et al. (2024)	Animal + cell	NGF-induced PC12 cell + PTZ epilepsy mice	✓	TNF-α, IL-1β, IL-6, IFN-γ	✓	–	✓	Microglia, astrocytes	✓	✓	✓	✓
Hooper et al. (2018)	Animal	Gabrd/Crh SE mice	–	–	–	✓	–	–	–	–	–	✓
Xia et al. (2022)	Animal	KA-induced SE mice	✓	IL-1β, IL-18	–	–	✓	Microglia (↑Iba-1), astrocytes	✓	–	–	–
He et al. (2024)	Animal	CUMS mice (C57BL/6)	✓	IL-6	✓	↑Corticosterone	✓	Microglia, hippocampal neurons	–	✓	✓	✓
Li et al. (2021)	Human	Drug-resistant TLE patients	–	–	–	–	✓	Neurons, microglia, astrocytes (↑GFAP)	–	–	–	✓
Ma et al. (2024)	Cell	Low-Mg^2+^-induced epileptiform discharges in hippocampal neurons	–	–	–	–	–	–	–	✓	✓	–

✓: Indicates the mechanism/phenomenon was observed or involved in the study.

–: Indicates the mechanism/phenomenon was not explicitly mentioned or evaluated in the study.

### 3.1 Inflammation-induced neurotransmitter dysregulation

Chronic inflammation significantly disrupts neurotransmitter balance in EDC, not only altering homeostasis but also affecting major neuromodulatory systems, including 5-HT, dopamine (DA), GABA, and glutamate (GLU). The primary inflammatory mechanisms contributing to the pathogenesis and progression of EDC primarily involve dysregulated tryptophan (Trp) metabolism and dysfunction of classical neurotransmitter systems.

#### 3.1.1 Tryptophan metabolism dysregulation and neurotransmitter imbalance

Dysregulation of Trp metabolism mediated by indoleamine 2, 3-dioxygenase 1 (IDO1) is a central mechanism underlying the development of EDC, as it impairs 5-HT synthesis and enhances neuronal excitability. Under inflammatory conditions, pro-inflammatory cytokines [e.g., interferon-gamma (IFN-γ)] can activate IDO1, diverting Trp metabolism toward the kynurenine (KYN) pathway while inhibiting 5-HT synthesis, resulting in serotonin depletion and exacerbation of neurotransmitter imbalance (Réus et al., 2015). Clinical studies (Deng et al., 2021) have shown an elevated KYN/Trp ratio in patients with depression, with similar metabolic disturbances observed in epilepsy, further suggesting a substantial role of this pathway in EDC. Quinolinic acid (QUIN), a Kyn metabolite and an N-methyl-D-aspartate (NMDA) receptor agonist, enhances glutamate excitotoxicity, promotes epileptiform discharges, and aggravates depressive behaviors. In contrast, kynurenic acid (KYNA), an NMDA receptor antagonist, exerts neuroprotective effects. Owever, in EDC, KYNA levels are reduced while QUIN accumulates excessively, representing a pathological shift that further exacerbates neurotransmitter dysregulation (Réus et al., 2015).

#### 3.1.2 Direct effects of inflammation on classical neurotransmitter systems

Beyond Trp metabolism, inflammation directly affects the synthesis, release, and receptor function of classical neurotransmitters, thereby exacerbating neural circuit dysfunction associated with EDC. Interleukin-1β (IL-1β) inhibits presynaptic 5-HT release and reduces dopaminergic neuronal activity. Abnormal Trp metabolism also contributes to excitatory/inhibitory (E/I) imbalance. In epileptic foci, IL-1β promotes GLU release from astrocytes and suppresses GABA synthesis, ultimately resulting in E/I imbalance (Tripathi et al., 2024). Animal studies have shown that excessive IL-1β disrupts calcium influx, impairs GABA-A receptor function, and leads to neuronal hyperexcitability (Zhu et al., 2006). TNF-α promotes Tumor Necrosis Factor Receptor 1 (TNFR1)-mediated internalization of GABA receptors, upregulates α-amino-3-hydroxy-5-methyl-4-isoxazolepropionic acid (AMPA)-type glutamate receptor expression, and enhances synaptic excitability, thereby triggering seizures and exacerbating emotional and cognitive dysfunction (Tripathi et al., 2024).

Anti-inflammatory therapy offers novel intervention strategies for EDC. Geraniol, a compound with anti-inflammatory and antioxidant properties, was shown to modulate GABAergic signaling, enhance GABA release, and inhibit GLU overactivation in a pentylenetetrazol (PTZ)-induced mouse model of epilepsy (Younis et al., 2024). This restored E/I balance and improved epilepsy-related cognitive dysfunction.

Chronic inflammation contributes to neurotransmitter imbalance by reducing levels of 5-HT, DA, and GABA while enhancing GLU excitotoxicity, thereby accelerating the progression of EDC. Inflammation-mediated Trp metabolic disruption, E/I imbalance, and neurotransmitter pathway dysfunction may represent central pathological mechanisms in the development of EDC.

### 3.2 Inflammation-induced dysregulation of the HPA axis

In EDC, a profoundly intertwined pathophysiological relationship exists between the HPA axis and neuroinflammation. Epileptic seizures can acutely activate the HPA axis, eliciting an immediate surge in glucocorticoids (e.g., cortisol). While this initial hormonal response may confer transient anti-inflammatory and neuroprotective effects, conditions of chronic stress or frequent seizures lead to a dysregulation of the HPA axis’s negative feedback mechanisms, culminating in persistent hypercortisolemia. This chronic glucocorticoid excess, in turn, promotes neuronal dysfunction and contributes to the pathogenesis of depressive-like behaviors (Juruena et al., 2020). Supporting this, Hooper et al. (2018) demonstrated in Gabrd/Crh mice that the absence of GABA receptor δ subunits on corticotropin-releasing hormone (CRH) neurons resulted in limited post-seizure corticosterone (CORT) elevation; these animals exhibited lower seizure frequency and reduced depressive behaviors, with effects reversible upon exogenous CORT administration. Moreover, sustained elevation of CORT can induce the upregulation of pro-inflammatory cytokines (e.g., IL-1β, IL-6, and TNF-α), enhance microglial activation, disrupt blood–brain barrier (BBB) integrity, and facilitate the infiltration of peripheral immune cells into the central nervous system (CNS), thereby exacerbating neuroinflammation (Hooper et al., 2018).

This cascade is consistent with an “inflammation-stress two-hit model” (Espinosa-Garcia et al., 2021). The “first hit” is characterized by chronic stress-induced release of glucocorticoids and High Mobility Group Box 1 (HMGB1). These mediators activate the microglial Toll-like receptor (TLR)/NF-κB pathway, leading to the transcriptional upregulation of NLRP3 inflammasome components and subsequently inducing a microglial primed state. The “second hit” is then precipitated by the epileptic seizure itself. This event triggers the primed microglia to assemble functional NLRP3 inflammasomes, which subsequently catalyze the maturation and substantial release of IL-1β, thereby further amplifying the inflammatory cascade (Vezzani and Baram, 2007). The synergistic action of these pro-inflammatory cytokines and glucocorticoids manifests as: (1) an enhancement of glutamatergic excitatory transmission coupled with suppression of GABAergic inhibitory tone, which promotes epileptic network synchronization; and (2) a disruption of neuroplasticity, including inhibited expression of neurotrophic factors like Brain-Derived Neurotrophic Factor (BDNF), contributing to the manifestation of depressive-like behaviors (Wu et al., 2020; Doherty et al., 2019). Consequently, HPA axis dysregulation, in conjunction with the sustained amplification of inflammatory responses, establishes a pernicious positive feedback loop.

### 3.3 Inflammation-induced immune cell activation

Aberrant immune activation, occurring both within the CNS and throughout the body’s peripheral systems, contributes directly to the intertwined pathologies of EDC. This section examines how dysregulated immune cells contribute to the complex interplay between seizures and depressive symptoms. This section also explores the distinct yet interconnected roles of central glial cells, such as microglia and astrocytes, and the broader peripheral immune system in amplifying the inflammatory cascade that characterizes EDC.

#### 3.3.1 Central immune system: aberrant activation of microglia and astrocytes

**Figure 1:**
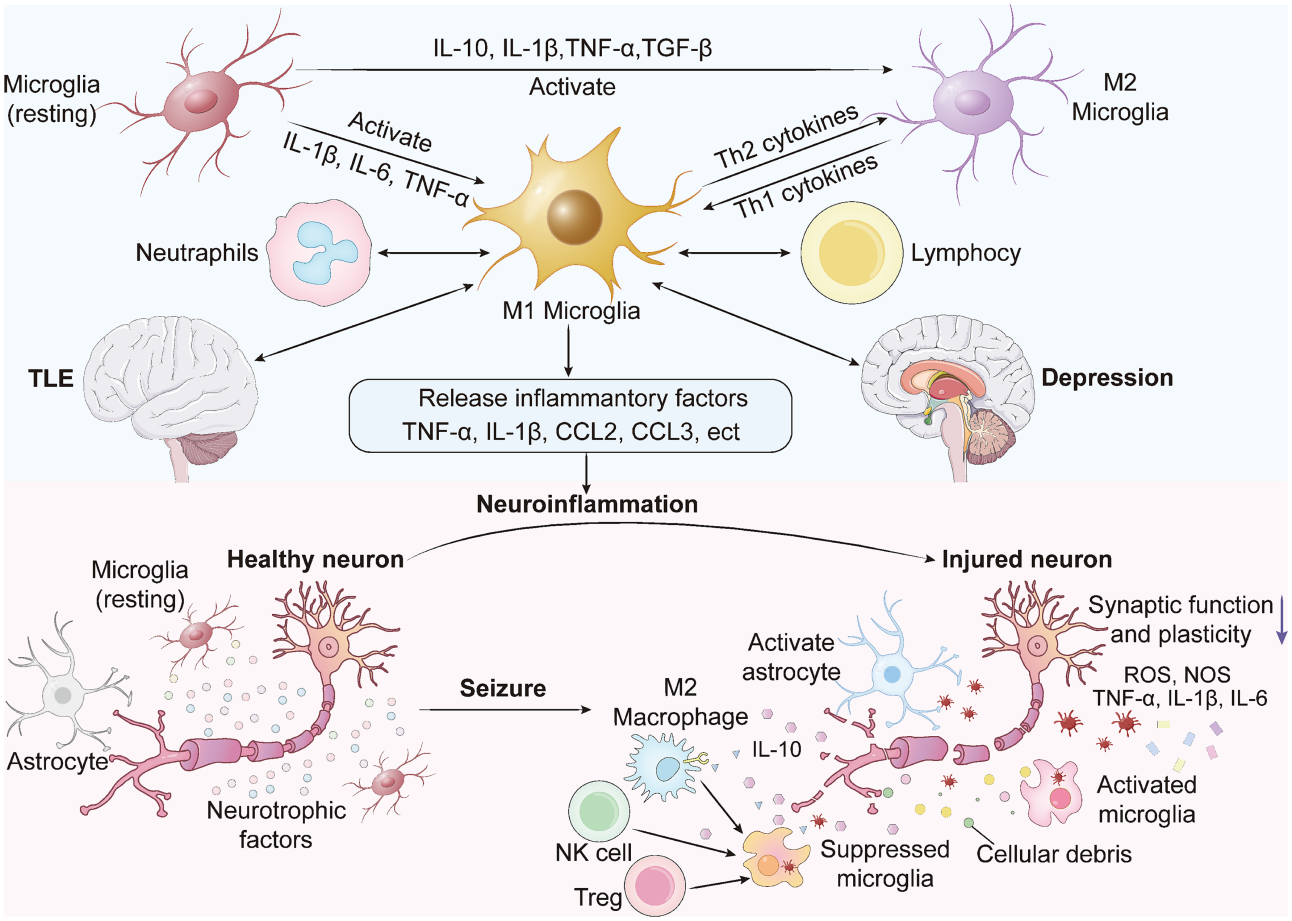
Bidirectional immune activation and neuroinflammation in EDC.

In EDC, aberrant microglial and astrocytic activation constitutes a central mechanism linking neuroinflammation to neural circuit dysfunction. As illustrated in Figure 1, seizures can transform resting-state microglia into either pro-inflammatory M1 or anti-inflammatory M2 phenotypes. M1 microglia exacerbate seizures and depressive symptoms by releasing pro-inflammatory cytokines, including C-X-C motif chemokine ligand 8 (CXCL8), IL-1β, IL-6, and TNF-α, which promote neuroinflammation and neuronal injury. Injured neurons, in turn, release additional inflammatory factors and reactive oxygen/nitrogen species (ROS/NOS), recruiting more activated microglia and perpetuating a vicious inflammatory cycle. Conversely, M2 microglia, regulatory T cells (Tregs), and natural killer (NK) cells can suppress M1 polarization and mitigate neuroinflammatory responses.

In stress-induced EDC models, significant activation of the NLRP3 inflammasome triggers microglial M1 polarization and initiates P2X purinoceptor 7 (P2X7R) pathway signaling. This contributes to the release of pyroptosis-related inflammatory cytokines and the disruption of BBB integrity (Wen et al., 2024). Concurrently, astrocytic dysfunction is also prominent in EDC. Astrocyte density is frequently observed to be downregulated in the prefrontal cortex and hippocampus of patients with depression. Dysregulated expression of astrocytic functional proteins, such as glutamate transporter-1 (GLT-1), aquaporin-4 (AQP4), and glutamine synthetase (GS), not only exacerbates mood disorders but also influences seizure thresholds (Gittins and Harrison, 2011). Insufficient glial function can lead to impaired glutamate clearance and diminished BBB support, thereby reducing neuroplasticity and precipitating mood disorders. Conversely, during epileptic seizures, glial cells become overactivated and participate in amplifying inflammation; elevated levels of various glia-derived proteins and cytokines in the cerebrospinal fluid (CSF) and blood of patients suggest that glia-mediated neuroinflammation persists throughout the course of epilepsy (Kalsariya et al., 2025). Furthermore, pro-inflammatory cytokines secreted by astrocytes within a chronic inflammatory milieu can also impact the serotonin system; for instance, IL-1β activates the Mitogen-Activated Protein Kinase (MAPK) pathway to enhance serotonin transporter (SERT) activity, leading to decreased synaptic 5-HT concentrations and thereby further inducing depressive-like behaviors (Zhu et al., 2010).

Collectively, microglia promote inflammatory cycles via the NLRP3 pathway, while astrocyte dysfunction exacerbates glutamate toxicity and BBB dysregulation. These glial contributions synergistically lead to the aggravation of seizures and depressive symptoms, constituting a critical neuroinflammatory amplification mechanism in EDC.

#### 3.3.2 Peripheral immune system: the role of chronic inflammation and immune imbalance

Extensive research indicates that epilepsy and depression may interact through inflammatory and immune pathways. Epileptic seizures and their associated chronic neurogenic stress can activate the peripheral immune system, leading to peripheral monocyte/macrophage activation and the release of pro-inflammatory cytokines, which subsequently influence the CNS (Mazumder et al., 2019). Peripheral inflammatory responses drive intracerebral inflammation via multiple routes, activating microglia and astrocytes to secrete pro-inflammatory mediators such as IL-1β, IL-6, and TNF-α (Mazarati et al., 2010; Hou et al., 2017). Epidemiological and clinical studies have shown that the high prevalence of depression in patients with epilepsy (up to 55%) is also associated with elevated inflammatory markers (Plevin and Smith, 2019; Lambert and Robertson, 1999). Current perspectives suggest a bidirectional interaction between peripheral immune activation and central inflammation: peripheral inflammation can enhance intracerebral inflammation via the BBB or vagal nerve pathways, while central inflammation can, in turn, stimulate the peripheral immune system (Shin et al., 2022; Miyata, 2022).

In the context of EDC, imbalances in immune cell subsets are of particular significance. Patients with depression often exhibit an increase in T helper 17 (Th17) cells and a decrease in Tregs, with inflammatory cytokines like IL-6 promoting Th17 differentiation while inhibiting Treg development (Sharifi et al., 2023). This Th17/Treg imbalance has also been observed in the periphery of patients with refractory epilepsy (especially in childhood epilepsy), suggesting that this immune skewing may participate in the pathogenesis of epilepsy and its associated depression (Alvarez-Mon et al., 2021; Li et al., 2010). Furthermore, the T helper 1/T helper 2 (Th1/Th2) immune axis typically shifts toward a pro-inflammatory Th1 bias: peripheral blood from depressed patients often shows elevated levels of Th1-type cytokines such as IFN-γ and interleukin-2 (IL-2), with a concomitant decrease in Th2 factors. This peripheral Th1 bias is associated with enhanced central inflammatory responses and may exacerbate the neuroinflammatory state in patients with epilepsy (Sharifi et al., 2023).

Peripheral pro-inflammatory cytokines affect the CNS through multiple mechanisms. Classical studies have demonstrated that cytokines such as interleukin-1 alpha/beta (IL-1α/β), IL-6, and TNF-α can cross the BBB into brain tissue via specific transport systems in circumventricular organs (CVOs) or other saturable mechanisms. These cytokines can also directly compromise BBB integrity (Miyata, 2022); for example, IL-1β can damage endothelial tight junctions, inhibit astrocytic production of Sonic Hedgehog (SHH) signaling, and induce the release of further pro-inflammatory factors, thereby exacerbating neuroinflammation (Bronisz et al., 2023). Additionally, peripheral inflammatory signals can be rapidly transmitted to the brain via pathways including the vagus nerve, activating the HPA axis and altering neurotransmitter metabolism. Animal studies have shown that in a rat model of TLE, elevated hippocampal IL-1β levels lead to HPA axis dysfunction and impaired hippocampal-raphe 5-HT pathway function, resulting in depressive-like behaviors (Mazarati et al., 2010); local administration of IL-1β receptor antagonists can significantly ameliorate these depressive-like phenotypes. This suggests that peripheral inflammatory cytokines like IL-1β may mediate the pathological processes of EDC through upstream neuroimmune pathways and alterations in neurotransmitters (Mazarati et al., 2009).

In the comorbid state of epilepsy and depression, peripheral and central inflammatory pathways can overlap and produce synergistic effects. Studies have found that compared to patients with epilepsy alone, those with comorbid depression exhibit higher peripheral blood levels of IL-1β, indicating a greater inflammatory burden in the dual-disease state (Shin et al., 2022). Furthermore, there is an intersection in the pathological mechanisms of epilepsy and depression: peripheral inflammation can intensify intracerebral inflammatory responses via neuro-immune pathways, promoting depressive phenotypes, while central inflammation, in turn, may lower the seizure threshold.

In summary, peripheral immune activation and central neuroinflammation exert synergistic effects in EDC. Cytokines such as IL-1β and IL-6 act as bridges between these compartments, transmitting peripheral inflammatory signals to the brain, where they are amplified by shared neuro-immune circuits. The interplay between these systems offers insights into novel therapeutic targets.

### 3.4 Pyroptosis and oxidative stress: converging inflammatory mechanisms in EDC

Pyroptosis and OS are synergistic inflammatory mechanisms that drive the pathophysiological processes of EDC. Within this context, the P2X7R-NLRP3-IL-1β pathway in microglia constitutes a central signaling axis. This axis integrates various intracellular stressors, including OS, ultimately mediating dysfunction in neural circuits.

The initiating event of this pathway involves the binding of adenosine triphosphate (ATP) [a damage-associated molecular pattern (DAMP)] released by neurons during epileptic seizures to microglial P2X7R. Activation of P2X7R plays a dual role: first, by inducing potassium ion (K^+^) efflux, it provides the necessary signal for NLRP3 inflammasome oligomerization and activation (Yue et al., 2017); second, by inhibiting GABA reuptake, it directly enhances network excitability (Barros-Barbosa et al., 2016). Mitochondria-derived ROS, as effector molecules of OS, serve as another upstream signal for NLRP3 activation, thereby forming a positive feedback loop between inflammation and excitability in EDC (Wu et al., 2025).

**Figure 2:**
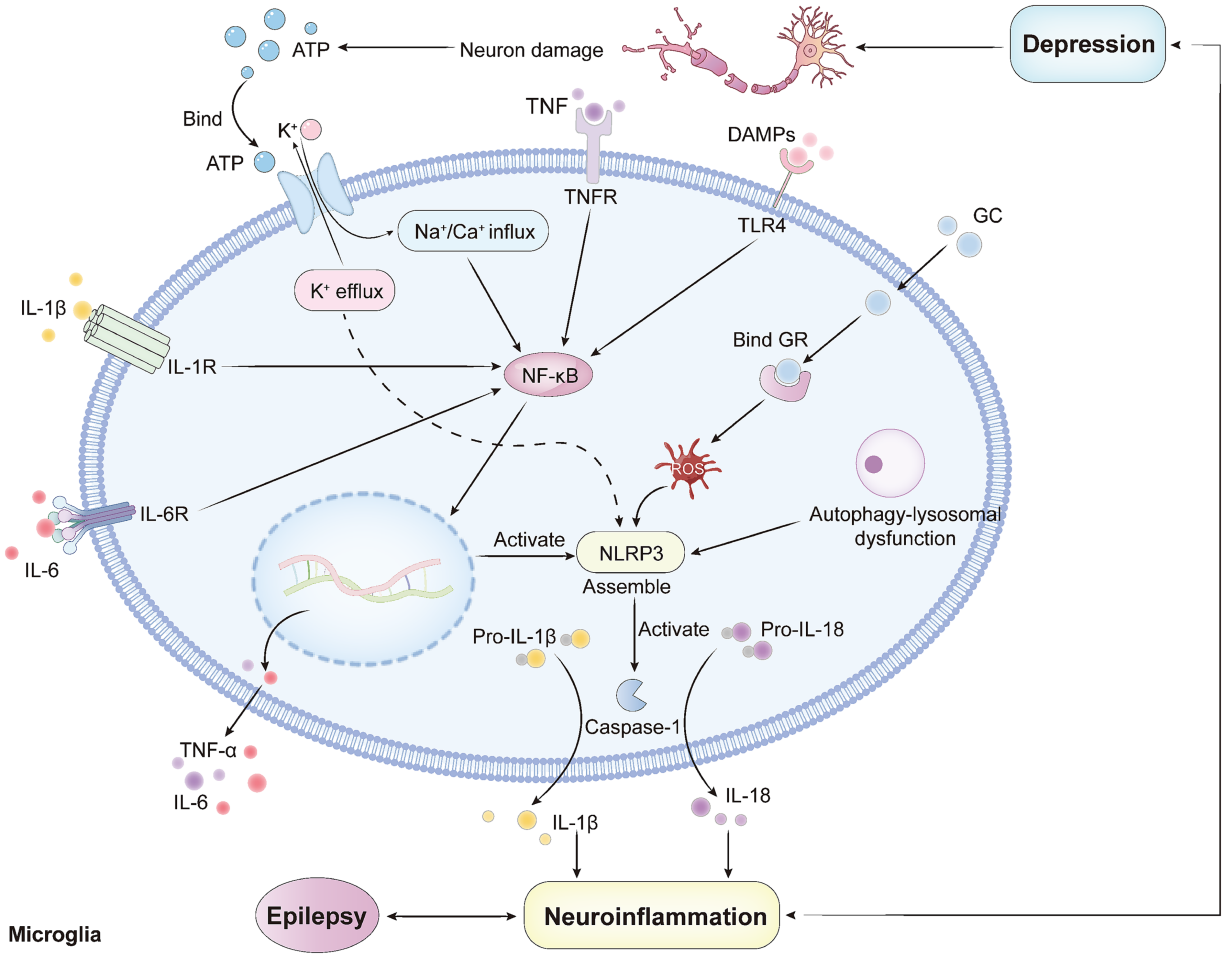
Neuroinflammatory signal transduction in microglia: mechanisms implicated in EDC.

The activated NLRP3 inflammasome acts as an enzymatic platform, recruiting and activating Caspase-1. Activated Caspase-1 then proteolytically cleaves Gasdermin D (GSDMD), releasing its pore-forming N-terminal fragment. This fragment oligomerizes into pores on the cell membrane, ultimately leading to pyroptosis (Xia et al., 2022). As illustrated in Figure 2, pyroptosis is a form of programmed cell death mediated by inflammasomes, in which activation of the NLRP3 inflammasome triggers caspase-1. This activation converts pro-interleukin-1β (pro-IL-1β) and pro-interleukin-18 (pro-IL-18) into their active forms, which are subsequently released extracellularly, initiating sustained inflammation that disrupts neural circuits and promotes both seizures and depressive behaviors. GSDMD-mediated glial cell pyroptosis compromises neural circuit integrity through two mechanisms: first, the loss of metabolic and ion homeostatic support for neurons due to cell death; and second, the bystander effect induced by molecules like IL-1β released from pyroptotic cells, including maladaptive synaptic remodeling and impaired synaptic plasticity (e.g., long-term potentiation, LTP) (Xia et al., 2022).

As an effector molecule downstream of this pathway, IL-1β exerts pleiotropic pathological effects. In epilepsy pathology, IL-1β not only directly enhances neuronal excitability but also acts on cerebrovascular endothelial cells. It impairs BBB permeability by inducing the degradation of tight junction proteins (e.g., occludin, claudin-5), thereby exacerbating neuroinflammation (Vezzani and Baram, 2007; Han et al., 2024). In the pathophysiology of depression, IL-1β synergistically contributes to central 5-HT system hypofunction: it activates IDO1, diverting Trp metabolism away from 5-HT synthesis, and upregulates the SERT to accelerate the clearance of 5-HT from the synaptic cleft (Zhu et al., 2006). Salidroside (a compound extracted from *Rhodiola rosea*) has been shown to alleviate depressive-like behaviors in chronically stressed mice by inhibiting P2X7/NF-κB/NLRP3 pathway-mediated pyroptosis (Xiao et al., 2022).

In summary, the P2X7R-NLRP3-GSDMD-IL-1β pathway constitutes a unified pathophysiological axis that integrates OS and pyroptosis signals. This pathway tightly couples the electrophysiological disturbances of epilepsy with the neurobiochemical imbalances of depression through multi-level molecular and cellular events. Molecular nodes within this pathway represent actionable targets for developing integrated therapeutic strategies for EDC.

### 3.5 Inflammation-induced impairment of synaptic plasticity

Overexpression of inflammatory cytokines reduces neurotrophic factor levels, thereby impairing synaptic plasticity and exacerbating neural circuit dysfunction and emotional dysregulation. In epilepsy, repeated seizures induce excitatory synaptic sprouting and loss of inhibitory synapses, ultimately leading to neural circuit reorganization. In particular, mossy fiber sprouting in hippocampal dentate granule cells lowers the seizure threshold and contributes to cognitive and emotional impairments (Hajszan and MacLusky, 2006). In contrast, depression is typically characterized by reduced neuroplasticity, including hippocampal atrophy, decreased dendritic spine density, and impaired neurogenesis (Doherty et al., 2019). These pathologies may act synergistically to exacerbate synaptic dysfunction in EDC.

Aberrant elevation of inflammatory cytokines—particularly IL-1β and TNF-α—represents a central pathological mechanism in EDC. IL-1β impairs synaptic plasticity by interfering with long-term potentiation (LTP), thereby affecting learning and memory processes. TNF-α regulates synaptic scaling, and its dysregulation compromises the stability of neural networks (Rizzo et al., 2018). Recent findings further support this mechanism, demonstrating that chronic unpredictable mild stress (CUMS) induces dysbiosis of the gut microbiota, which in turn activates central inflammatory responses. This leads to upregulation of pro-inflammatory cytokines and microglial activation in the hippocampus, ultimately suppressing the generation of Bromodeoxyuridine-positive/Doublecortin-positive (BrdU+/DCX+) immature neurons and Bromodeoxyuridine-positive/Neuronal Nuclei-positive (BrdU+/NeuN+) mature neurons. The resulting impairment in hippocampal neurogenesis disrupts synaptic remodeling and contributes to sustained depressive-like behaviors, underscoring the pivotal role of inflammation-mediated synaptic plasticity deficits in CUMS-induced depression (He et al., 2024).

Inflammation also modulates synaptic function through signaling pathways such as BDNF–Two-pore-domain potassium channel TREK-1 (TREK-1) and Phosphoinositide 3-kinase/Akt/mechanistic target of rapamycin (PI3K/Akt/mTOR). Overexpression of TREK-1 suppresses BDNF-mediated plasticity, and its immunoreactivity is significantly higher in TLE-D patients than in those with TLE alone, suggesting a role in inflammation-mediated neuronal damage (Li et al., 2021). The PI3K/Akt/mTOR pathway is another critical target of inflammatory modulation (Lu et al., 2023). In epilepsy models, pro-inflammatory cytokines downregulate this pathway via phosphatase and tensin homolog (PTEN)-mediated suppression, thereby reducing neuronal survival and synaptic efficacy. Suppression of inflammation has been shown to partially restore PI3K/Akt/mTOR signaling and improve neuronal function (Ma et al., 2024).

Further studies have demonstrated that the immunosuppressant mycophenolate mofetil significantly reduces IL-1β and IL-2 expression, reverses associated pathological changes, and improves behavioral outcomes in TLE-D animal models (Mazumder et al., 2019). However, the inflammatory impact on synaptic plasticity may be dose-dependent and bidirectional. High-dose administration of either pro-inflammatory agents [e.g., Lipopolysaccharide (LPS)] or anti-inflammatory drugs (e.g., ibuprofen) impairs neuroplasticity, whereas low-dose treatments produce minimal adverse effects (Golia et al., 2019). These findings suggest that maintaining a dynamic balance of inflammation may be essential for optimizing treatment strategies in EDC.

## 4 PART 3. Peripheral–central inflammatory crosstalk: the role of the gut–brain axis

The pathogenesis of EDC extends beyond the CNS, with the GBA acting as a bidirectional conduit that links peripheral and central inflammatory responses. The GBA consists of multiple interconnected systems, including the gut microbiota (GM), enteric nervous system, vagus nerve, immune system, and neuroendocrine signaling.

The gut microbiota, a diverse microbial community residing within the host, functions as a critical component of the GBA. It can influence brain function through multiple pathways, thereby participating in the pathophysiology of EDC (Morkl et al., 2023). Under physiological conditions, the GM modulates brain activity via nutrient metabolism, immune regulation, and neurotransmitter synthesis. However, gut dysbiosis is frequently observed in patients with epilepsy and depression, which impairs intestinal barrier integrity and allows microbial products to enter the systemic circulation, triggering systemic inflammation and contributing to CNS dysfunction (Abavisani et al., 2024; Zhu et al., 2024).

Fecal microbiota transplantation (FMT) from patients with inflammatory depression has been shown to induce both peripheral and central inflammation in mice, leading to depression-and anxiety-like behaviors. In contrast, supplementation with *Clostridium butyricum* and specific probiotics can restore microbial homeostasis, reduce inflammatory cytokines, and ameliorate depressive-like behaviors (Liu et al., 2024). Furthermore, patients with inflammatory bowel diseases (IBD)—including Crohn’s disease and ulcerative colitis—have an increased risk of developing both epilepsy and depression, often accompanied by pronounced microbial imbalance and elevated pro-inflammatory cytokines (Shaikh et al., 2020; Ding et al., 2021).

Compared with conventional antidepressant treatments, microbiota-targeted therapies (e.g., probiotics) have shown more promising outcomes in mitigating mood disturbances. Long-term use of ASMs has also been linked to gut microbial dysbiosis; reduced abundance of *Bifidobacterium* spp. has been observed in fecal samples from patients with drug-resistant epilepsy (Shaikh et al., 2020). These findings suggest that the gut microbiota acts as an essential mediator linking peripheral and central inflammation in the progression of EDC.

The GBA regulates CNS function through three primary pathways: immune, neural, and neuroendocrine/metabolic signaling, thereby establishing a dynamic feedback loop between systemic inflammation and brain activity.

### 4.1 Immune pathway

The gut microbiota modulates the host immune system and influences central neuroinflammation by regulating peripheral inflammatory status. Dysbiosis disrupts the intestinal epithelial barrier, facilitating the translocation of bacterial products—such as LPS—into the bloodstream and triggering systemic inflammation, a process referred to as endotoxemia. LPS, as a strong pro-inflammatory stimulus, activates the monocyte–macrophage system to release cytokines such as TNF-α and IL-1β, contributing to the development of chronic low-grade inflammation (Shaikh et al., 2020). In an epilepsy-prone environment, this LPS-driven peripheral inflammation markedly lowers the seizure threshold. Animal experiments have confirmed that even low-dose LPS exposure can exacerbate the severity of pro-epileptic stimuli (such as febrile seizures or chemical convulsants), accompanied by an increase in brain inflammatory markers like COX-2 and IL-1β (Ho et al., 2015; Kovács et al., 2014). Regarding depression, LPS exposure can induce typical “sickness behavior,” including anhedonia and reduced activity, which is consistent with clinically observed post-infection depression (Bufalino et al., 2013). In the context of EDC, elevated LPS levels caused by gut microbiota dysbiosis can be considered a “third hit.” Building upon existing central pathologies, this “third hit” exacerbates neuroinflammation, synergistically worsening both epileptic seizures and depressive symptoms, thereby perpetuating a vicious cycle (Arora et al., 2022).

Moreover, chronic LPS exposure has been shown to lower the seizure threshold, activate brain glial cells, and induce depressive-like behaviors in animal models (Chen et al., 2025). These findings suggest that the gut microbiota may promote the onset and progression of EDC through the propagation of inflammatory signals that activate both peripheral and central immune responses.

### 4.2 Neural pathway

The vagus nerve is an essential component of the GBA, responsible for conveying metabolic signals from the gut microbiota to the CNS and regulating brain function. Studies have shown that the vagus nerve can sense gut microbiota-derived metabolites and modulate central neurotransmitter levels through neuroendocrine signaling (Shaikh et al., 2020). Metabolites produced by *Lactobacillus* spp. can activate mucosal receptors in the gut, with signals transmitted via the vagus nerve to the brain, thereby exerting anxiolytic and antidepressant effects.

This dual function of the vagus nerve impacts the pathology of EDC. On one hand, it mediates the “cholinergic anti-inflammatory pathway,” which involves inhibiting the production of inflammatory factors by peripheral macrophages through the release of acetylcholine, thereby controlling systemic inflammation (Li et al., 2024). This is essential for mitigating epilepsy-and depression-related inflammatory damage. Experimental evidence shows that the neuroprotective effects of probiotics disappear after vagotomy, directly demonstrating the gut microbiota’s reliance on the vagus nerve to modulate central inflammation (Ding et al., 2021). On the other hand, when severe intestinal inflammation is present, the vagus nerve can also transmit these harmful inflammatory signals (such as gut-released IL-1β) to the CNS, inducing microglial activation (Rosas-Ballina et al., 2011). Therefore, modulating vagal nerve activity (e.g., through clinical vagus nerve stimulation (VNS) therapy) is a viable strategy for intervening in EDC.

Bravo et al. reported that *Lactobacillus administration* attenuated stress-induced CORT release via the HPA axis and improved anxiety- and depression-like behaviors in mice. These effects were abolished following vagotomy, highlighting the essential role of the vagus nerve in mediating the mood-regulating effects of probiotics (Bravo et al., 2011).

Beyond emotional regulation, the vagus nerve has also been shown to suppress seizure activity by attenuating neuroinflammation. VNS in mouse models of IBD comorbid with epilepsy has been shown to reduce intestinal inflammation and decrease seizure severity (Ding et al., 2021; Chen et al., 2025). These findings underscore that the gut microbiota can transmit inflammatory signals to the brain via the vagus nerve, thereby influencing the pathogenesis and progression of EDC.

### 4.3 Endocrine and metabolic pathways

The gut microbiota can affect CNS function both directly and indirectly by producing neuroactive metabolites and modulating hormone levels. Short-chain fatty acids (SCFAs), such as butyrate and propionate, are metabolites produced by anaerobic gut bacteria that can cross the BBB and provide energy to neurons. Butyrate, in particular, binds to receptors on astrocytes, suppresses neuroinflammation, enhances neurotrophic factor expression, and reduces seizure frequency in epileptic rats (Citraro et al., 2021). However, SCFA levels are significantly reduced in WAG/Rij rats, a genetic model of absence epilepsy, which may compromise GBA-mediated immune homeostasis and worsen EDC symptoms (Citraro et al., 2020).

The immunomodulatory functions of SCFAs are particularly noteworthy. They can bind to free fatty acid receptors (FFARs) on immune cells, while butyrate, as a HDAC inhibitor, can upregulate the expression of anti-inflammatory genes and induce microglial polarization toward an anti-inflammatory phenotype (Zhao et al., 2022; Cheng et al., 2024). This anti-inflammatory property has been confirmed to alleviate neuroinflammation and behavioral abnormalities in epilepsy and stress-induced depression models.

In terms of neurotransmitter regulation, the gut microbiota exerts multi-faceted influences. Many symbiotic bacteria (e.g., *Lactobacillus, Bifidobacterium*) can directly synthesize neuroactive substances like GABA and 5-HT precursors. Additionally, SCFAs can indirectly affect central neurotransmitter balance; for instance, butyrate can enhance the efficiency of tryptophan entering the brain for 5-HT synthesis by modulating the HPA axis (Cheng et al., 2024). Therefore, insufficient production of SCFAs and neurotransmitter precursors due to gut dysbiosis directly contributes to the pathological state of uncontrolled neuroinflammation and neurotransmitter imbalance in EDC.

The gut microbiota also contributes to neuroendocrine regulation by influencing the synthesis of neurotransmitters such as 5-HT, thereby modulating CNS function. Over 90% of the body’s serotonin is synthesized in the gut, and certain gut bacteria—such as *Clostridium* and *Streptococcus*—can produce 5-HT precursors or even bioactive serotonin itself. Dysbiosis may reduce peripheral 5-HT availability, thereby decreasing central 5-HT levels and exacerbating depressive symptoms (Margolis et al., 2014). In addition to serotonin, the gut microbiota can also modulate the balance of excitatory and inhibitory neurotransmitters. Probiotic supplementation has been shown to increase the GABA/GLU ratio, thereby alleviating EDC-related neuropathology and restoring neurotransmitter homeostasis (Mazzoli and Pessione, 2016).

## 5 PART 4. Inflammation-related therapeutic strategies

Given the central role of inflammation in the pathogenesis and progression of EDC, inflammation-targeted interventions have become a growing area of research in recent years. Many studies have investigated a variety of strategies aimed at alleviating inflammation and improving EDC-related symptoms. This section summarizes six major therapeutic strategies based on inflammatory mechanisms, in light of the latest research advances. These include anti-inflammatory agents, antioxidants, gut microbiota modulation, herbal medicine and natural compounds, acupuncture-based interventions, and precision therapies. These approaches offer novel insights into the therapeutic management of EDC.

### 5.1 Anti-inflammatory agents

Anti-inflammatory therapy plays a significant role in EDC by targeting pro-inflammatory mediators in both the peripheral immune system and the CNS, thereby improving disease progression, reducing seizure risk, and alleviating depressive symptoms. Glucocorticoids, such as methylprednisolone, are widely used in the treatment of neuroimmune disorders due to their potent anti-inflammatory properties. Studies have shown that a three-month course of intravenous methylprednisolone pulse therapy significantly reduces seizure frequency, improves VSMS, and improves electroencephalographic parameters, with no serious adverse effects reported. These findings further support its safety and efficacy in the treatment of EDC (Rangarajan et al., 2022).

With ongoing research, precision anti-inflammatory strategies targeting EDC-associated pro-inflammatory factors have garnered growing interest. Among these, COX-2 and the NLRP3 inflammasome have emerged as promising therapeutic targets. Celecoxib, a highly selective COX-2 inhibitor, has been shown to suppress neuronal hyperexcitability and reduce seizure frequency in pilocarpine- and kainate-induced epilepsy models, suggesting its potential therapeutic utility in epilepsy management (Lim et al., 2019) In addition, MCC950, an NLRP3 inflammasome inhibitor, has demonstrated significant neuroprotective effects in animal studies by reducing neuronal damage after status epilepticus (SE) and alleviating depressive-like behaviors, representing a novel avenue for precision anti-inflammatory therapy (Hong et al., 2024).

Beyond systemic immune modulation, agents that regulate CNS-specific neuroinflammation have also demonstrated promise. The CB2 receptor (CB2R) agonist AM1241, an anti-neuroinflammatory compound, modulates the NLRP3 inflammasome and AMP-activated protein kinase (AMPK) signaling pathways to suppress microglial overactivation, thereby reducing seizure frequency and alleviating depressive-like symptoms (Cai et al., 2024). Minocycline, a known microglial inhibitor, has been reported to decrease inflammatory cytokine levels and improve behavioral outcomes in animal models of epilepsy and depression. However, its clinical efficacy remains controversial and requires further investigation (Vayanakkan et al., 2024).

### 5.2 Antioxidants

OS plays a central role in EDC by inducing mitochondrial dysfunction, promoting the release of pro-inflammatory cytokines, and altering synaptic plasticity, thereby exacerbating seizures and worsening depressive symptoms (Becker et al., 2019). Antioxidants such as melatonin (MT) and NAC have been shown to exert neuroprotective effects by mitigating OS-related damage in the context of EDC.

MT, as an endogenous antioxidant, is capable of crossing the BBB, scavenging free radicals, and upregulating the expression of antioxidant enzymes such as superoxide dismutase (SOD) and glutathione peroxidase (GPx), thereby enhancing antioxidant capacity (Banach et al., 2011). In addition to directly suppressing OS, MT inhibits pyroptosis mediated by the NLRP3/GSDMD pathway, reduces inflammation, and activates the nuclear factor erythroid 2-related factor 2 (Nrf2) signaling pathway to strengthen endogenous antioxidant defenses, ultimately exhibiting both anticonvulsant and antidepressant effects (Tchekalarova et al., 2020).

NADPH oxidase (NOX) is a key enzyme responsible for ROS production, playing critical roles in cellular signaling, immune regulation, and inflammatory responses. However, chronic NOX overactivation can disrupt redox balance, leading to synaptic damage, neuronal apoptosis, and cognitive deficits, thereby contributing to the progression of EDC. Apocynin, a NOX inhibitor, has been shown to reduce seizure-induced ROS overproduction, suppress neuroinflammation, improve cognitive function, and alleviate anxiety- and depression-like behaviors in preclinical models (Jaiswal and Kumar, 2022).

As a precursor of glutathione (GSH), NAC restores antioxidant capacity, decreases neuroinflammation, and has demonstrated beneficial effects in EDC. NAC has been shown to reduce depressive-like behaviors in WAG/Rij epileptic rats, potentially through GSH-mediated attenuation of oxidative injury (Dean et al., 2011). However, excessive NAC administration may paradoxically trigger seizures, potentially due to its modulatory effects on the glutamate–cysteine antiporter system. Thus, the optimal dosing and therapeutic window for NAC in EDC warrants further investigation.

Antioxidants not only reduce seizure frequency and improve depressive symptoms but also exert therapeutic effects by attenuating neuroinflammation, enhancing synaptic plasticity, and restoring neurotransmitter homeostasis, offering a multifaceted approach to modulate the pathophysiology of EDC.

### 5.3 Microbiota-targeted interventions

Gut microbiota dysbiosis has been recognized as a central pathological mechanism in EDC. Modulating gut microbial composition has been shown to improve neuroinflammation, regulate neurotransmitter homeostasis, and contribute positively to the alleviation of EDC symptoms (Shaikh et al., 2020; Ding et al., 2021).

Probiotic supplementation, as a microecological intervention, primarily functions by replenishing beneficial strains such as Lactobacillus to restore intestinal homeostasis. Probiotics exert both neurotrophic and anti-inflammatory effects and have been associated with improvements in clinical outcomes in EDC. Recent experimental evidence further supports these findings, showing that FMT from stress-resilient mice significantly increased the abundance of beneficial genera such as *Lactobacillus* and *Bifidobacterium*, alleviated depressive-like behaviors, and improved performance in behavioral tests. In parallel, FMT promoted hippocampal neurogenesis, as indicated by increased numbers of BrdU+/DCX + and BrdU+/NeuN+ neurons. Notably, these antidepressant effects were abolished when neurogenesis was pharmacologically blocked, suggesting that the therapeutic benefits of probiotic-driven microbiota modulation are closely dependent on the restoration of neurogenesis and synaptic plasticity (He et al., 2024). Clinical studies have further demonstrated that long-term probiotic supplementation can reduce seizure frequency in patients with TLE and improve anxiety and depression scores (Wang et al., 2022).

The emerging concept of “psychobiotics” refers to specific probiotic strains (e.g., *Bifidobacterium longum, Lactobacillus* spp.) that exert antidepressant and anxiolytic effects. Their mechanisms may involve enhancing peripheral Trp levels, promoting the production of SCFAs, and modulating vagal tone (Kim et al., 2022).

In addition to probiotics, FMT has been explored as an alternative strategy to reestablish microbial balance by transferring fecal material from healthy donors. Studies have shown that WAG/Rij rats exhibit intestinal villus disruption and inflammatory infiltration even 1 month prior to the onset of seizures. FMT has been found to effectively restore intestinal morphology, restructure microbial composition, and reduce absence seizure episodes (Citraro et al., 2021).

Dietary modulation also plays a role in shaping the gut microbiome. The ketogenic diet (KD), characterized by high fat, low carbohydrate, and adequate protein content, has been widely used in epilepsy management. In addition to its anticonvulsant effects, KD has been reported to confer neurotrophic, antioxidant, neuroprotective, and anti-inflammatory benefits, contributing to seizure reduction and alleviation of depressive symptoms (Operto et al., 2020).

Taken together, the gut microbiota has emerged as a promising therapeutic target in EDC. Microbiota-targeted interventions modulate peripheral and central inflammation, stabilize neurotransmitter signaling, and offer a novel pathway for modifying disease progression.

### 5.4 Herbal medicines and natural bioactive compounds

Herbal medicines and natural compounds have demonstrated significant therapeutic potential in the management of EDC due to their multi-component, multi-target, and multi-pathway synergistic effects. Among these, gastrodin, the primary bioactive compound of *Gastrodia elata*, exhibits anti-inflammatory, antioxidant, and neurotransmitter-modulatory properties. Studies have shown that water extracts of gastrodin inhibit overactivation of the NLRP3 inflammasome via activation of the extracellular signal-regulated kinase 1/2 (ERK1/2)–Nrf2 signaling pathway, thereby suppressing neuroinflammatio. Additionally, gastrodin has been reported to modulate gut microbiota composition and restore neurotransmitter balance, alleviating chronic stress-induced depressive symptoms and cognitive dysfunction (Huang et al., 2021).

Berberine (Ber), a broad-spectrum anti-inflammatory and antioxidant agent, has also shown significant neuroprotective effects in EDC. It reduces ROS levels, enhances the activity of SOD and GPx, and inhibits the recruitment of microglia and neutrophils, thereby decreasing the expression of pro-inflammatory cytokines such as TNF-α, IL-1β, and IL-6. These effects collectively contribute to increased seizure thresholds and improved depressive symptoms (Shou and Shaw, 2022). Ber also regulates gut microbiota by increasing the abundance of “psychobiotic” species, such as *Bifidobacterium* and *Lactobacillus*, thereby stabilizing neurotransmitter systems and exerting antidepressant effects (Küpeli Akkol et al., 2021).

In addition to single compounds, herbal formulas offer broader therapeutic coverage through their synergistic effects. Xiaoyao San (XYS), a classical herbal formula commonly used for emotional regulation in traditional Chinese medicine, contains a core herb pair (*Bupleurum-Paeonia*) that has been shown to attenuate oxidative stress, modulate HPA axis activity, and exert neuroprotective effects, leading to symptom improvement in depressive rat models (Lv et al., 2022). Further studies indicate that XYS inhibits the TLR4/NLRP3 signaling pathway, thereby reducing colonic inflammation in CUMS rats (Liu et al., 2024). Given the bidirectional role of the GBA, this peripheral anti-inflammatory effect may be transmitted centrally, contributing to antidepressant outcomes.

Beyond herbal interventions, certain dietary components have also been found to exert neuroprotective, anti-inflammatory, and antioxidant effects. Walnuts, rich in polyphenols, have been shown to suppress epilepsy-induced neuroinflammation, inhibit microglial activation, enhance synaptic plasticity, and maintain GABA/GLU balance, thus improving cognitive and emotional deficits associated with epilepsy (Ghasemzadeh Rahbardar and Hosseinzadeh, 2024). Capsaicin, through activation of transient receptor potential vanilloid 1 (TRPV1), inhibits neuroinflammation, promotes the release of neurotrophic factors, and regulates central neurotransmitter levels, thereby alleviating symptoms of both epilepsy and depression (Pasierski and Szulczyk, 2022).

Taken together, herbal medicines and natural compounds offer multifaceted therapeutic actions in EDC by attenuating inflammation and oxidative stress, modulating neurotransmitter systems, optimizing HPA axis function, and maintaining GBA homeostasis.

### 5.5 Acupuncture

Acupuncture, a traditional Chinese medical therapy, has been shown to improve neuroimmune imbalance, inflammatory status, and neurotransmitter dysregulation in patients with EDC. Systematic reviews suggest that acupuncture modulates neurotrophic factors (e.g., BDNF), neurotransmitters (e.g., 5-HT, dopamine), inflammatory cytokines, and the neuroendocrine system, exerting both antidepressant and anticonvulsant effects (Huang et al., 2024).

Further preclinical studies have demonstrated that acupuncture at Baihui (GV20), Yintang (EX-HN3), and other related acupoints significantly inhibits microglial polarization toward the pro-inflammatory M1 phenotype and reduces the expression of TNF-α and IL-1β by suppressing the Toll-like receptor 4 (TLR4)/Myeloid differentiation primary response 88 (MyD88)/NF-κB signaling cascade, thereby attenuating neuroinflammation (Jiang et al., 2024). In addition to direct anti-inflammatory effects, acupuncture has also been shown to regulate gut microbiota composition and improve GBA function. Specifically, electroacupuncture downregulates histone deacetylase 2, promoting microbial homeostasis and reducing the abundance of pro-inflammatory taxa, ultimately mitigating neuroinflammation and alleviating pain and depressive-like behaviors in chronic stress models (Li et al., 2024).

Moreover, acupuncture attenuates hyperactivation of the HPA axis, thereby improving endocrine imbalance under chronic stress conditions. Acupuncture has been shown to reduce levels of CRH, adrenocorticotropic hormone (ACTH), and CORT, suppressing stress-induced neurotransmitter imbalances and inflammatory responses (Bravo et al., 2011). Acupuncture not only intervenes in the pathological processes of EDC but also acts synergistically with conventional pharmacotherapy. Studies have found that acupuncture enhances the efficacy of ASMs (e.g., valproic acid), reduces adverse effects, and improves treatment tolerance (Xu et al., 2022).

Beyond EDC, acupuncture has also demonstrated clinical potential in addressing psychiatric comorbidities such as anxiety and insomnia in patients with epilepsy. Taken together, acupuncture exerts multifaceted therapeutic effects on EDC by modulating neuroinflammation, optimizing HPA axis activity, restoring GBA homeostasis, and regulating neurotransmitter systems.

### 5.6 Precision neuroprotective strategies

Precision medicine refers to a therapeutic strategy that targets disease-specific mechanisms based on individualized pathological features, with the goal of achieving enhanced efficacy and safety. Among emerging approaches, MSCs have gained traction for their potential in treating epilepsy and its neuropsychiatric comorbidities. MSCs are multipotent cells characterized by self-renewal, multilineage differentiation, and immunomodulatory capabilities. Owing to their ability to cross the BBB, regulate neuroinflammation, and repair damaged tissues, MSCs have emerged as promising candidates in the treatment of neurodegenerative diseases and epilepsy-related conditions.

MSCs exert neuroprotective effects by secreting anti-inflammatory cytokines and growth factors that help reestablish immune homeostasis. In animal models of epilepsy, MSC transplantation has been shown to reduce neuroinflammatory markers and neuronal loss (Salari et al., 2020). Moreover, MSCs release exosomes—extracellular vesicles enriched with microRNAs (miRNAs) and proteins—that serve as essential mediators of intercellular communication. These exosomes have been shown to target microglia and astrocytes, suppress inflammatory responses, and enhance synaptic plasticity, suggesting promising applications in EDC (Xu et al., 2024).

Further experimental studies have demonstrated that intranasal administration of MSC-derived exosomes significantly improved cognitive deficits in mice with SE, possibly by modulating neuroinflammation and enhancing neuroplasticity (Long et al., 2017). In addition to their anti-inflammatory effects, MSCs have also been implicated in antioxidative activity, neurotransmitter regulation, and BBB repair, all of which may contribute to pathological improvements in EDC.

The utility of MSCs in epilepsy and neuropsychiatric comorbidities has been confirmed in multiple studies. For instance, Senthilkumar et al. used a kainic acid-induced TLE model to assess BBB disruption following SE and identified the optimal time window for intravenous infusion of dental pulp stem cells (DPSCs) and bone marrow–derived mesenchymal stem cells (BM-MSCs) (Senthilkumar et al., 2023). Although only a small number of engrafted MSCs were detected in regions such as the corpus callosum and subgranular zones 3 months after infusion, systemic delivery of DPSCs/BM-MSCs significantly attenuated neurodegeneration and neuroinflammation and improved depressive-like behaviors in TLE rats (Senthilkumar et al., 2023). These findings suggest that the therapeutic effects of MSCs may rely more on paracrine signaling or immune modulation rather than direct cellular integration.

Encouraging progress has also been made in the safety evaluation of MSC-based therapy. A Phase I clinical trial for drug-resistant epilepsy has evaluated the safety and preliminary efficacy of MSC transplantation, reporting good tolerability and no serious adverse events, thereby supporting MSCs as a promising therapeutic strategy for EDC (Hlebokazov et al., 2021).

## 6 Conclusions and perspectives

This review highlighted the pivotal role of inflammation in EDC and discussed inflammation-targeted therapeutic strategies, including anti-inflammatory agents, antioxidants, gut microbiota modulation, herbal and natural products, acupuncture, and stem cell therapy. Current evidence suggests that inflammation contributes to EDC through upregulation of central inflammatory cytokines, overactivation of microglia and astrocytes, and hyperactivation of the HPA axis, leading to neurotransmitter imbalance, impaired synaptic plasticity, increased pyroptosis, and OS—positioning EDC as a prototypical inflammation-driven and stress-driven neuropsychiatric comorbidity (Xu et al., 2018; Chen et al., 2014). Furthermore, the GBA plays an essential role in the pathophysiology of EDC. Dysbiosis of the gut microbiota can trigger systemic immune activation and influence the brain through both the circulatory system and the vagus nerve, thereby promoting neuroinflammation and aggravating seizure and depressive symptoms (Bravo et al., 2011; An et al., 2016). Recent studies have demonstrated that modulation of the gut microbiota can reduce seizure frequency, alleviate depressive symptoms, and suppress neuroinflammatory responses, suggesting that GBA-targeted interventions represent a promising therapeutic direction for EDC (He et al., 2024).

Although the role of neuroinflammation in EDC has been widely investigated, several limitations persis. First, the causal relationship between inflammation and the onset and progression of EDC remains unclear, as large-scale longitudinal studies evaluating whether inflammatory markers can serve as early predictors of depression risk in epilepsy patients are currently lacking. Second, heterogeneity in inflammatory subtypes may exist among EDC patients, with some individuals exhibiting IL-1β -driven immune responses, while others may show predominance of TNF-α, HMGB1, or NLRP3 inflammasome activation; however, a validated biomarker-based classification system is currently lacking. Moreover, although preclinical studies support the efficacy of anti-inflammatory interventions in ameliorating epilepsy and comorbid depression, clinical trial results remain heterogeneous, suggesting that inflammation suppression alone may be insufficient to fully control disease progression.

Future research on EDC should prioritize a deeper understanding of inflammatory mechanisms, the development of precision medicine-based interventions, exploration of innovative therapies, and optimization of integrated management strategies. First, long-term follow-up studies are needed to clarify the temporal dynamics of inflammation in EDC and to identify inflammatory biomarkers that may serve as early prognostic indicators. Second, advanced technologies such as single-cell transcriptomics and proteomics are needed to define inflammation subtypes and enable individualized anti-inflammatory treatments. Innovative therapeutic avenues include enhancing the brain-targeted delivery of natural compounds (e.g., curcumin, resveratrol) to improve their anti-inflammatory and antioxidant efficacy, and engineering MSCs to continuously release anti-inflammatory factors for early-stage intervention in epileptogenesis. Finally, multimodal strategies—such as combining anti-inflammatory agents with psychological interventions—warrant evaluation to determine whether multi-targeted approaches outperform monotherapies.

In conclusion, inflammation spans the entire course of EDC and serves as a central interface linking peripheral and central systems, as well as neural, immune, and endocrine pathways. With continued elucidation of inflammatory mechanisms and advances in emerging therapies, precision medicine holds promise for delivering individualized treatment for EDC, potentially reducing comorbidity rates and improving long-term patient outcomes. These inflammation-targeted strategies may also offer insights into novel neuroprotective approaches to mitigate aging-related neuronal injury.
